# Economic benefits of methylmercury exposure control in Europe: Monetary value of neurotoxicity prevention

**DOI:** 10.1186/1476-069X-12-3

**Published:** 2013-01-07

**Authors:** Martine Bellanger, Céline Pichery, Dominique Aerts, Marika Berglund, Argelia Castaño, Mája Čejchanová, Pierre Crettaz, Fred Davidson, Marta Esteban, Marc E Fischer, Anca Elena Gurzau, Katarina Halzlova, Andromachi Katsonouri, Lisbeth E Knudsen, Marike Kolossa-Gehring, Gudrun Koppen, Danuta Ligocka, Ana Miklavčič, M Fátima Reis, Peter Rudnai, Janja Snoj Tratnik, Pál Weihe, Esben Budtz-Jørgensen, Philippe Grandjean

**Affiliations:** 1 EHESP School of Public Health, Rennes Cedex, France; 2 FPS Health, Food Chain Safety and Environment, Brussels, Belgium; 3 Karolinska Institutet, Stockholm, Sweden; 4 Instituto de Salud Carlos III, Majadahonda, Madrid, Spain; 5 National Institute of Public Health, Prague, Czech Republic; 6 EDI, Bundesamt für Gesundheit, Liebefeld, Switzerland; 7 Health Service Executive South, Cork, Ireland; 8 Laboratoire National de Santé, Luxembourg, Luxembourg; 9 Environmental Health Center, Cluj-Napoca, Romania; 10 Public Health Authority of the Slovak Republic, Bratislava, Slovakia; 11 Cyprus State General Laboratory, Nicosia, Cyprus; 12 Department of Public Health, University of Copenhagen, Copenhagen, Denmark; 13 Umweltbundesamt, Berlin, Germany; 14 Flemish Institute for Technological Research, Mol, Belgium; 15 Nofer Institute of Occupational Medicine, Lodz, Poland; 16 Jožef Stefan Institute, Ljubljana, Slovenia; 17 Faculdade de Medicina de Lisboa, Lisboa, Portugal; 18 National Institute of Environmental Health, Budapest, Hungary; 19 Faroese Hospital System, Tórshavn, Faroe Islands; 20 Institute of Public Health, University of Southern Denmark, Odense, Denmark; 21 Department of Environmental Health, Harvard School of Public Health, Boston, MA, USA

**Keywords:** Economic evaluation, Methylmercury, Prenatal exposure, Neurodevelopmental deficits

## Abstract

**Background:**

Due to global mercury pollution and the adverse health effects of prenatal exposure to methylmercury (MeHg), an assessment of the economic benefits of prevented developmental neurotoxicity is necessary for any cost-benefit analysis.

**Methods:**

Distributions of hair-Hg concentrations among women of reproductive age were obtained from the DEMOCOPHES project (1,875 subjects in 17 countries) and literature data (6,820 subjects from 8 countries). The exposures were assumed to comply with log-normal distributions. Neurotoxicity effects were estimated from a linear dose-response function with a slope of 0.465 Intelligence Quotient (IQ) point reduction per μg/g increase in the maternal hair-Hg concentration during pregnancy, assuming no deficits below a hair-Hg limit of 0.58 μg/g thought to be safe. A logarithmic IQ response was used in sensitivity analyses. The estimated IQ benefit cost was based on lifetime income, adjusted for purchasing power parity.

**Results:**

The hair-mercury concentrations were the highest in Southern Europe and lowest in Eastern Europe. The results suggest that, within the EU, more than 1.8 million children are born every year with MeHg exposures above the limit of 0.58 μg/g, and about 200,000 births exceed a higher limit of 2.5 μg/g proposed by the World Health Organization (WHO). The total annual benefits of exposure prevention within the EU were estimated at more than 600,000 IQ points per year, corresponding to a total economic benefit between €8,000 million and €9,000 million per year. About four-fold higher values were obtained when using the logarithmic response function, while adjustment for productivity resulted in slightly lower total benefits. These calculations do not include the less tangible advantages of protecting brain development against neurotoxicity or any other adverse effects.

**Conclusions:**

These estimates document that efforts to combat mercury pollution and to reduce MeHg exposures will have very substantial economic benefits in Europe, mainly in southern countries. Some data may not be entirely representative, some countries were not covered, and anticipated changes in mercury pollution all suggest a need for extended biomonitoring of human MeHg exposure.

## Background

Methylmercury (MeHg) is a well-documented neurotoxicant, and prenatal exposures are therefore of particular concern [1,2]. The main sources of exposure are seafood and freshwater fish [3]. Thus, MeHg exposures vary with dietary habits, contamination levels, and species availability. While the distribution of MeHg exposures has been studied in substantial detail in the United States [4], only scattered information is available on MeHg exposures in Europe.

Because the critical effect of MeHg exposure is developmental brain toxicity, exposures among women of reproductive age groups are of primary concern [5,6]. As has previously been determined in regard to lead exposure [7], developmental MeHg exposure is linked to a loss in Intelligence Quotient (IQ), with associated lower school performance and educational attainment, thereby leading to long-term impacts on societal benefits of pollution abatement [8]. These consequences may be expressed in terms of economic impacts, as has been demonstrated in United States [9,10]. However, few economic evaluations have been performed in Europe [8,11,12], primarily because of the lack of exposure data.

Based on harmonised protocols developed in COPHES [13], the DEMOCOPHES project has just completed a multi-country study of hair-mercury concentrations in women of reproductive age groups in 17 European countries. In conjunction with literature data, we now utilise the exposure data to generate estimates of economic impacts of MeHg exposures in Europe.

The economic assessment relies on several assumptions. The hair-Hg concentrations is used as the main exposure indicator in this study, and any blood-based measurements also considered are expressed in terms of hair-mercury using a conversion factor of 250 [14,15]. In regard to the dose-response function (DRF), a linear model is usually the default [14], although it may not necessarily provide the best statistical fit to the data [16]. We therefore used the linear slope as the primary DRF and then conducted a sensitivity analysis using the log function, where each doubling of exposure above the background causes the same deficit of 1.5 IQ points [10].

With regard to background exposures and the possible existence of a threshold, the U.S. EPA’s Reference Dose (RfD) of 0.1 μg/kg body weight/day corresponds to a hair-Hg concentration of about 1 μg/g hair [14]. Updated calculations [17] resulted in an adjusted biological limit about 50% below the recommended level, corresponding to 0.58 μg/g hair. The validity of this lower cut-off point below the RfD is supported by recent studies of developmental neurotoxicity at exposure levels close to the background [18-21]. We assumed that, below the 0.58 μg/g cut-off point, only negligible adverse effects would exist. As additional reference point, we use a tolerable limit proposed by the World Health Organization (WHO), which corresponds to a hair-Hg concentration of approximately 2.5 μg/g [22]. This limit takes into account the possible compensation of MeHg toxicity by beneficial nutrients in seafood [22].

## Methods

### Exposure information

DEMOCOPHES is a cross-sectional survey of European population exposure to environmental chemicals. The human exposure biomarkers included the hair-mercury concentration and was collected in 17 European countries based on children aged 6–11 years and their mothers. A common European protocol, developed by the COPHES project, was followed in each country. The main inclusion and exclusion criteria were (1) residence in the study area for at least five years, and (2) not having metabolic disturbances. The period of sampling was September 2011 to February 2012. A total of 1,875 child-mother pairs were recruited from urban and rural communities in the participating countries, while excluding exposure hot-spots. Major efforts were carried out to achieve high quality and comparability of data. Standard operational procedures for total mercury concentrations in hair were developed and validated by the Laboratory of Environmental Toxicology in Spain, to ensure comparable measurements, which included a strict quality assurance programme, in which seventeen European laboratories participated. Each DEMOCOPHES partner contributed information to allow estimation of the underlying distribution of exposures in the population, where rural and urban results were merged. In addition, each partner provided the frequencies of results above the cut-off levels of 0.58 μg/g, 1.0 μg/g, and 2.5 μg/g. The latter corresponds to WHO’s tolerable limit, which takes into account likely toxicity compensation by beneficial nutrients in seafood [22].

Additional information on MeHg exposures in Europe was obtained to complement the DEMOCOPHES data. Thus, information of similar quality was extracted from published articles (Miklavčič, unpublished data), and distribution information from comparable studies was obtained from Belgium, Denmark, France, Norway, Slovenia, and the United Kingdom. As explained below, missing information was calculated assuming a log-normal distribution of the exposures.

### Exposure distributions

Using the number of births in 2008 and the observed hair-Hg concentrations, we estimated the number of births exceeding the three exposure limits for each country and obtained the sum for all of the EU. For missing EU member states, MeHg exposures were assumed to be the same as a neighbouring country. The year 2008 was chosen as the closest to the time during which the exposure data had been collected, and it allowed complete information for the calculations envisaged. Due to the existence of sampling uncertainty, “smoothed” proportions exceeding the three limits were calculated assuming log-normal distributions. Because log-transformed concentrations would follow a normal distribution, the parameters in the log-normal distributions could be estimated by standard normal distribution methods. Each data set included probabilities (prob) for being below specific percentiles (perc). The parameters in the logarithmic distributions were therefore obtained as the intercept and slope when regressing log(perc) on Φ^-1^(prob), where Φ is the cumulative distribution function of the standard normal distribution. Using the total numbers of births in 2008, numbers of births exceeding the three cut-off limits in each country were calculated from observed and smoothed distributions.

### Calculation of IQ benefits

A linear dose-response function was applied as the default model [14]. Thus, as a 1 μg/L increase of the cord-blood mercury concentration is associated with an average adverse impact on IQ of 0.093 times the standard deviation (which is standardised to be 15), each increase in the maternal hair-mercury by 1 μg/g is associated with an average loss of 0.465 IQ points [10]. This slope is based on a range of neuropsychological tests and subtests administered in the Faroe Islands study at age 7 years [23]. As some recent studies [18-21] suggest MeHg-associated deficits close to or below the cut-off level of 0.58 μg/g hair, the calculations may represent an underestimate. In addition, the slope may be steeper at low exposure levels. Thus, a log model was used for sensitivity analyses. In this model, a doubling in prenatal MeHg exposures is associated with a delay in development of 1.5–2 months at age 7, which corresponds to about 10% of the standard deviation, i.e. 1.5 IQ points [1]. Again, we applied this slope for exposures above the 0.58 μg/g the cut-off point.

To estimate the benefits at exposures above the cut-off point, we calculated the average hair-mercury concentration in women exceeding 0.58 μg/g based on 1,000,000 simulations from the estimated log-normal distribution (as described above). After deduction of the 0.58 μg/g and multiplication by the slope factor, an average IQ benefit was obtained. This amount was then multiplied by the annual number of births exceeding the cut-off level. A similar calculation was made in the logarithmic dose-response model except that here we calculated the average log-transformed mercury concentration in women exceeding 0.58 μg/g, deducted log(0.58) and multiplied by the slope factor of the logarithmic dose-response model (1.5/log(2)).

### Annual benefits of exposure reduction

The major component of the social costs incurred by an IQ reduction is loss of productivity and thus a lower earning potential [9,24]. The economic consequence of prenatal exposure to MeHg is valued as the lifetime earning loss per person. We assumed singleton births only, so that the number of women was equal to the cohort size. We also assumed that IQ deficits present at age 7 years or preschool ages are permanent [25]. The estimated individual benefits are the avoided lifetime costs using 2008 data (slightly lower benefits are obtained if referring to more recent years, and benefits are only minimally affected by subsequent membership of the Euro zone). The benefit estimates originate from the 2008 figure of €17,363 per IQ point as recently calculated for France based on data from the United States [24]. For the various European countries involved, this value is adjusted for differences in purchasing power. While simple currency exchange conversion and Gross Domestic Product (GDP) per capita do not adjust for price differences, Purchasing Power Parity (PPP) conversion rates allow for comparison based on a common set of average international prices [26,27]. We also carried out the calculations after adjustment for productivity as the ratio of PPP-adjusted real GDP/capita in each country in relation to the US as a reference. The estimated value of an IQ point then takes into account the impact of labour costs and productivity (Additional file 1).

## Results

Table 1 and Additional file 2 show summary information on MeHg exposures in the European countries covered by DEMOCOPHES or other exposure studies. There is a clear trend from north and east to southern countries, most likely due to differences in dietary habits and availability of large fish species from the Mediterranean (the sources of exposure were not considered in the present study). In Table 1, exposures in Austria were assumed to be similar to those in Germany, as suggested by available data [28]. Exposure information from the Flemish part of Belgium [29,30] do not differ much from the national data obtained in DEMOCOPHES, which were therefore used for the calculations. The Flemish data were used to represent exposures in The Netherlands. In the absence of exposure data from Estonia, Finland, Latvia, and Lithuania, the DEMOCOPHES exposure information from Sweden was applied. National data from France are available [31] and have been used in recent economic calculations [8]. Data for Croatia and Greece were obtained from a recent birth cohort study [32]. Two exposure studies had been carried out in Italy, one in the northeast [32] and one in Naples [33], and a joint distribution was therefore used to obtain national exposure distributions that would also apply to Malta. Thus, a log-normal distribution was first fitted to each Italian data subset, and then the parameters of a joint log-normal distribution were determined as the mean of the parameters for the two distributions. Recent results from the Norwegian national birth cohort were used for this country [34]. As DEMOCOPHES data from the United Kingdom covered only a small rural sample, we relied on data on blood-mercury in pregnant women obtained from the ALSPAC birth cohort study in the 1990s [35]. Additional exposure data from Ukraine [36] supported the notion that MeHg exposures in Eastern Europe are low, with only small percentages exceeding the cut-off level, but this study was considered too small to be used for detailed calculations. The same applied to several other sources identified (Miklavčič, unpublished data).

**Table 1:** **Annual numbers of births and numbers exceeding three cut**-**off limits**, **as indicated by hair**-**mercury analyses** (**in μg/g**) **in population samples in European countries**

**Country**^**a**^	**Annual number of births** (**2008**)	**Number of samples**^**b**^	**Above 0**.**58 μg/g**		**Above 1**.**0 μg/g**		**Above 2**.**5 μg/g**	
			**Proportion in sample (%)**	**Estimated number of births**	**Proportion in sample (%)**	**Estimated number of births**	**Proportion in sample (%)**	**Estimated number of births**
Austria	77,800	NA	(6.7)	5,213	(0.8)	622	(0)	0
Belgium	127,200	129	28.7	36,506	9.3	11,830	0	0
		242^c^	23.2	29,510	7.2	9,158	0	
Bulgaria	77,700	NA	(4.2)	3,263	(1.2)	932	(0.8)	622
Croatia	43,800	234^d^	52.0	22,776	22.0	9,636	4.7	2,059
Cyprus	9,200	60	36.7	3,376	18.3	1,684	3.3	304
Czech Republic	119,600	120	5.0	5,980	0.8	957	0	0
Denmark	65,000	145	36.6	23,790	13.1	8515	0.7	455
Estonia	16,000	NA	(10.0)	1,600	(2.0)	320	(0)	0
Faroe Islands	675	505^e^	62.6	423	30.2	204	5.3	36
Finland	59,500	NA	(10.0)	5,950	(2.0)	1,190	(0)	0
France	829,300	126^f^	44.0	364,892	14.51	120,331	0.61	5,059
Germany	682,500	120	6.7	45,728	0.8	5,460	0	0
Greece	118,300	454^d^	78	92,274	57	67,431	14	16,562
Hungary	99,100	120	0.83	823	0	0	0	0
Ireland	74,000	120	10.8	7,992	2.5	1,850	0	0
Italy	576,700	891^d^ + 115^g^	(65.6)	378,315	(36.8)	212,226	(5.7)	32,872
Latvia	23,834	NA	(10.0)	2,383	(2.0)	477	(0)	0
Lithuania	35,100	NA	(10.0)	3,510	(2.0)	702	(0)	0
Luxembourg	5,600	55	32.7	1,831	18.2	1,019	0	0
Malta	4,100	NA	(65.6)	2,690	(36.8)	1,509	(5.7)	234
Netherlands	184,600	NA	(23.2)	42,827	(7.2)	13,291	(0)	0
Norway	60,500	119^h^	27.7	16,759	5.9	3,570	0	0
Poland	414,500	120	1.7	7047	0	0	0	0
Portugal	104,600	120	90.8	94,977	57.5	60,145	8.3	8,682
Romania	221,900	120	4.2	9,320	1.2	2,663	0.8	1,775
Slovakia	57,400	129	5.43	3,117	0.8	459	0	0
Slovenia	21,800	156	22.0	4,796	7.7	1,679	1.9	414
Spain	519,800	120	88.5	460,023	74.2	385,692	31.7	164,777
Sweden	109,300	100	10.0	10,930	2.0	2,186	0	0
Switzerland	76,700	120	5.0	3,835	2.1	1,611	0	0
United Kingdom	794,400	4134^h^	31.0	246,264	5.1	40,200	0	0
Total EU (27)	5,400,000			1,865,416		903,169		231,754

Exposures in EU countries without recent data are estimated from neighbouring countries (modelled results not based on observed distributions are given in parenthesis).

^**a**^ For countries without available exposure data (for number of samples, NA denotes not available), data from a neighbouring country have been applied to allow EU-wide estimates, and frequencies are given in parenthesis. This applies to Austria (data from Germany were used), Bulgaria (Romania), Netherlands (Flanders [30]), and Estonia, Finland, Latvia, and Lithuania (Sweden); ^b^ All data are from DEMOCOPHES, unless otherwise noted; ^c^[30]; ^d^[32]; ^e^ Pal Weihe, unpublished data; ^f^[31]; ^g^[33]; ^h^ Jean Golding, pers.comm.

The estimated number of annual births in the EU that exceed the 0.58 μg/g cut-off is about 1.8 million (Table 1, Additional file 3). The EPA limit is exceeded in about 900,000 births, and the WHO limit in 200,000 births within the EU. As each study is subject to sampling uncertainty, log-normal distribution models showed similar, though sometimes slightly higher, proportions exceeding the 0.58 cut-off level (Table 2). The data from Eastern European countries and from Croatia, the Faroe Islands, Norway, and Switzerland suggest that, within Europe, the great majority of births exceeding the various limits occur in EU member states.

**Table 2:** **Annual number of births with excess exposure**, **average hair**-**Hg concentration**, **IQ benefit from prevention of excess exposure**, **and the value of the IQ benefits**

**Country**	**Number of births above 0**.**58 μg/g**		**Average concentration above 0**.**58 μg/g**	**Benefit in IQ points**		**Value of 1 IQ point (Euro)**	**Total benefit (million Euro)**	
	**Modelled**	**Observed**		**Modelled**	**Observed**		**Modelled**	**Observed**
Austria	3,812	5,213	0.917	597	817	16,044	9.6	13.1
Belgium	39,686	36,506	0.939	6,625	6,094	16,458	109.0	100.3
Bulgaria	3,186	3,263	1.455	1,296	1,328	7,529	9.8	10.0
Croatia	21,769	22,776	1.355	7,845	8,208	11,320	88.8	92.9
Cyprus	3,514	3,376	1.311	1,195	1,148	13,747	16.4	15.8
Czech Republic	5,143	5,980	0.847	639	742	10,797	6.9	8.0
Denmark	22,815	23,790	1.027	4,742	4,945	20,220	95.9	100.0
Estonia	1,840	1,600	0.846	228	198	10,339	2.4	2.0
Faroe Islands	406	423	1.323	140	146	20,220	2.8	2.9
Finland	6,843	5,950	0.846	846	736	17,288	14.6	12.7
France	405,528	364,892	0.989	70,186	69,397	17,363	1,218.6	1,204.9
Germany	33,443	45,728	0.917	5,241	7,166	15,292	80.1	109.6
Greece	94,403	92,274	1.563	50,131	49,000	13,201	661.8	646.9
Hungary	892	823	0.884	126	116	9,691	1.2	1.1
Ireland	7,104	7,992	0.946	1,209	1,360	17,927	21.7	24.4
Italy	378,315	(378,315)	1.045	81,801	(81,801)	17,062	1,395.7	(1,395.7)
Latvia	2,741	2,383	0.846	339	295	11,568	3.9	3.4
Lithuania	4,037	3,510	0.846	499	434	9,661	4.8	4.2
Luxembourg	1,870	1,831	1.212	550	538	17,062	9.4	9.2
Malta	2,690	(2,690)	1.045	582	(582)	11,111	6.5	6.5
Netherlands	45,227	42,827	0.909	6,919	6,552	15,857	109.7	103.9
Norway	16,759	16,759	0.866	2,237	2,229	20,051	44.8	44.7
Poland	6,218	7,047	0.751	494	560	9,979	4.9	5.6
Portugal	94,349	94,977	1.482	39,573	39,836	12,221	483.6	486.8
Romania	9,098	9,320	1.455	3,702	3,797	8,187	30.3	31.1
Slovakia	2,468	3,117	0.899	366	462	10,037	3.7	4.6
Slovenia	4,840	4,796	1.194	1,382	1,369	11,939	16.5	16.3
Spain	479,775	460,023	2.136	347,137	332,845	13,558	4,706.5	4,512.7
Sweden	12,570	10,930	0.846	1,555	1,352	17,167	26.7	23.2
Switzerland	6,520	3.835	0.902	976	574	18,346	17.9	10.5
United Kingdom	248,647	246,200	0.81	26,593	26,338	15,324	407.5	403.5
EU Total	1,926,652	1,865,365		654,551	639,804		9,458	9,256

Data are for European countries with information on methylmercury exposure distributions. For countries without detailed observed data available, the modelled results are given in parenthesis. Sources of underlying data are as in Table 1.

Table 2 presents the estimated IQ losses associated with the MeHg exposures using the linear model, along with the estimates of economic impacts. We used both the observed data and the modelled distributions, and only small differences were seen, thus supporting the notion that the log-normal exposure distribution has an appropriate fit. The greatest benefits accrue for the largest countries with the highest proportions of subjects with exposures above the cut-off level. The total benefit from control of MeHg exposure was the highest for Spain and the lowest for Hungary. On a per capita basis, the calculated benefits are the greatest in the Faroe Islands and the southern countries, Spain, Greece, Portugal, Italy, and Croatia. The total annual benefits in terms of IQ points within the EU were estimated to be in excess of 600,000 per year for the linear DRC. With an average benefit of €13,579 per IQ point, the total economic benefits are estimated to exceed €9,000 million per year. When adjustment for productivity is included, the benefits are somewhat lower for several countries, and the EU total is slightly less than €8,000 million per year (Additional file 3).

For comparison, Table 3 shows the estimated IQ losses and economic benefits using the log transformed DRF. Due to the steeper curve shape at exposures close to the cut-off point of 0.58 μg/g, the estimated benefits are about 4-fold greater, at about 2.7 million IQ points per year, which correspond to total benefits for the EU of approximately €39,000 million or, after productivity adjustment, €33,000 million.

**Table 3:** **Annual number of births with excess exposure**, **the average log hair**-**Hg concentration**, **and IQ benefit and value from prevention of excess exposure** (**logarithmic dose**-**effect relationship**)

**Country**	**Number of births above 0**.**58 μg**/**g**	**Average log concentration above 0**.**58 μg**/**g**	**Benefit in IQ points**	**Value of 1 IQ point** (**Euro**)	**Total benefit** (**million Euro**)
Austria	3,812	−0.157	3,199	16,044	51.3
Belgium	39,686	−0.128	35,790	16,458	589.0
Bulgaria	3,186	0.128	4,638	7,529	34.9
Croatia	21,769	0.142	32,350	11,320	366.2
Cyprus	3,514	0.109	4,972	13,747	68.3
Czech Republic	5,143	−0.216	3,658	10,797	39.5
Denmark	22,815	−0.060	23,932	20,220	483.9
Estonia	1,840	−0.214	1,317	10,339	13.6
Faroe Islands	406	0.139	600	20,220	12.1
Finland	6,843	−0.214	4,897	17,288	84.7
France	405,528	−0.053	368,742	17,363	6,402.5
Germany	33,443	−0.157	28,060	15,292	429.1
Greece	94,403	0.355	183,808	13,201	2,426.4
Hungary	892	−0.186	692	9,691	6.7
Ireland	7,104	−0.132	6,345	17,927	113.7
Italy	378,315	−0.036	416,490	17,062	7.106.2
Latvia	2,741	−0.214	1,962	11,568	22.7
Lithuania	4,037	−0.214	2,889	9,661	27.9
Luxembourg	1,870	0.053	2,419	17,062	41.3
Malta	2,690	−0.036	2,961	11,111	32.9
Netherlands	45,227	−0.155	38,144	15,857	604.8
Norway	16,759	−0.198	12,574	20,051	252.1
Poland	6,218	−0.312	3,131	9,979	31.2
Portugal	94,349	0.277	167,777	12,221	2,050.4
Romania	9,098	0.128	13,245	8,187	108.4
Slovakia	2,468	−0.173	1,986	10,037	19.9
Slovenia	4,840	0.034	6,061	11,939	72.4
Spain	479,775	0.561	1,148,026	13,558	15,564.9
Sweden	12,570	−0.214	8,996	17,167	154.4
Switzerland	6,520	−0.167	5,329	18,346	97.8
United Kingdom	248,647	−0.244	161,816	15,324	2,479.7
EU Total	1,884,563		2,645,953		39,061

Data from European countries, sources of underlying data are as in Table 1.

## Discussion

This study provides for the first time regional European data on economic benefits of controlling MeHg exposure in relation to prevention of developmental neurotoxicity. It relies on data from a multi-country study of hair-Hg concentrations with a high level of quality assurance and with similar population sampling criteria. In addition, available data from other studies have been taken into consideration to provide supplementary information, thereby allowing EU-wide estimates to be calculated. Given the low MeHg exposures in Eastern Europe and the relatively small contributions from Croatia, the Faroe Islands, Norway, and Switzerland, the results suggest that benefits for all of Europe will not be substantially above the benefits calculated for the EU.

Several assumptions and caveats must be acknowledged. The hair-Hg concentration is an established biomarker of human MeHg exposure and is generally considered reliable [14]. We used available data from DEMOCOPHES and other sources, with most studies including only about 120 subjects. The sampling size and strategy may have underestimated the occurrence of uncommon high-level exposures, which would weigh more in the calculation of IQ benefits. Adjustment for this bias is obtained in the modelled distributions, which tended to show slightly greater benefits. Although these calculations rely on an assumption of a log-normal distribution of the exposures, the concurrence of the two sets of estimates support the validity of this assumption.

In calculating the IQ benefits, we used a linear dose-response function for the decrease in IQ at increased prenatal MeHg exposures, and this curve shape is an approximation of unknown validity. As has been documented for lead [37], a logarithmic DRF may be plausible, and a log curve shows a slightly better fit [16]. As the results for the log curve (Table 3) are about 4-fold higher than those obtained for the linear curve, the benefits calculated in Table 2 must be considered likely underestimates. In recent calculations using French data using similar methods [8], the logarithmic curve shape also resulted in substantially higher estimates.

The cut-off level assumed to be 0.58 μg/g hair may also result in underestimated benefits. Recent data from Poland [20], Japan [21] and the United States [18,19] suggest that a lower threshold is likely. If the threshold is indeed lower than we have assumed, the benefits of controlling MeHg exposures will likely be greater, although an additional effort may be required to achieve such lower exposures. Further, given that the much higher tolerable limit of 2.5 μg/g is likely exceeded by 200,000 births in the EU per year, clear benefits will accrue already from controlling the very highest exposures.

The IQ benefits from controlling mercury pollution were translated into economic impacts based on the calculated current life-time income benefits from a higher IQ level. These benefits are mainly based on studies carried out in the United States [24,38], and it is possible that IQ-linked differences in life-time incomes may not be the same in Europe. Adjustment for differences in purchasing power has been included to take this issue into partial account. We used data from 2008 to secure complete data sources; the use of more recent records would change the estimates only slightly. An alternative approach might be to calculate benefits from prevention of specific diseases, e.g. for mental retardation or autism, associated with MeHg exposure. However, the attributable risks associated with increases in MeHg exposure are unknown, and such calculations are therefore uncertain [10,39].

Some sources of imprecision in exposure estimates must be emphasized. Thus, in several cases when exposure information was not available for an EU member state, data from a neighbouring country were used as a proxy. Further, the results reported in DEMOCOPHES and in published reports may not be representative for each country. Although high fish consumers may possibly have been oversampled, it is more likely that the avoidance of known exposure hot-spots resulted in lowered exposure estimates. In addition, especially for small studies, an element of uncertainty exists with regard to the frequencies of the highest exposures, although this problem was addressed by modelling a log-normal distribution of exposures. Temporal variation and time trends may also play a role, especially in regard to older data. We have assumed stable diets, so that any seasonal or other time trends as well as the time dependence of MeHg sensitivity during brain development would not matter for the calculation of impacts.

Our focus on the loss in life-time earnings is similar to the avoidable costs previously calculated in relation to lead exposure [24]. Other costs were ignored, such as direct medical costs linked to treatment or interventions for children with neurodevelopmental disorders. We also neglected indirect costs, such as those related to special education or additional years of schooling for children as a consequence of these disorders, as well as intangible costs. In addition, our study did not consider other avoided direct health care costs in the longer term, such as those potentially related to the treatment of cardiovascular or neurodegenerative effects of MeHg exposure, which could be important for high fish consumers [2], but would be difficult to estimate. Any compensation of the IQ benefit due to special education and other remedies was not taken into account. Overall, the estimates presented in Table 2 are likely underestimates of the total benefits of MeHg exposure abatement.

Clear differences are apparent between European countries. Seafood and freshwater fish constitute the main source of exposure, but countries with high fish consumption levels, such as Spain and Norway, clearly show great differences in MeHg exposure that are undoubtedly related to the choice of fish species consumed as well as the contamination level. The high exposure levels observed in Spain are in accordance with other studies [40,41]. The elevated exposures in the Faroes are likely related to the occasional consumption of pilot whale meat [23].

Calculations from the United States have resulted in several greatly varying estimates, depending on the DRF assumptions. One comparable estimate put the aggregate economic benefit for each annual birth cohort in the US at $8.7 billion (range: $0.7–$13.9 billion for year 2000) [10]. We recently calculated the annual benefit for the US at about 264,000 IQ points, which would correspond to benefits of approximately $5 billion [42]. The EU benefits of over 600,000 IQ points are much higher. However, in comparing the figures for the US and the EU, note should be taken that annual number of births in the EU (5.4 million) are 27% greater than the 4.2 million births in the US per year. In addition, MeHg exposures in parts of Europe are higher than in the US [4]. On a global scale, benefit estimates can be extended on the basis of GDP values adjusted for PPP and productivity, but the validity of such calculations is limited by the lack of exposure assessments [43]. However, the present study leaves little doubt that global benefits substantially exceed $20 billion.

The present study did not aim at calculating annual costs of investments in pollution abatement due to the paucity of available data. Relevant investment costs would consider mercury emissions controls in coal-fired power plants, reduction of mercury usage in the chlorine industry, measures taken in dentistry, plus expenses for recycling and treatment of mercury releases. Some information is available and suggests that one-time expenses may be quickly balanced by the cumulated annual benefits from exposure abatement [9]. However, mercury emissions control needs to be carried out on a global level due to the regional and hemispherical dispersion of mercury releases [43]. These costs would likely have additional socioeconomic yields from better control of mercury emissions, e.g. job creation and modernization of capital equipment.

The control of inorganic mercury emissions will only result in diminished MeHg exposure in the long term, and the benefits will therefore be delayed. As MeHg exposure mainly originates from seafood and freshwater fish, public health advice on dietary choices is an important element of the intervention [6,44]. Due to the essential nutrients present in seafood [3], a reduction in MeHg exposure should not be sought through a decrease or replacement of fish in the diet. A prudent advice would be to maintain fish consumption and minimise the MeHg exposure by consumption of fish known to have lower MeHg concentrations, e.g., smaller species, younger fish, and catches from less polluted waters. Such advice should be directed toward women during pregnancy as the most cost-effective preventive action. Restricted consumption of large, piscivorous fish species may also benefit overfished populations of pelagic fish, such as tuna [45].

The successful completion of the DEMOCOPHES project and the complements from other exposure studies in Europe illustrate the feasibility and usefulness of biological monitoring approaches, in particular when relying on hair samples that may be easily obtained, stored and transported. While such studies have become a routine function in the United States through the National Health And Nutrition Examination Survey [4], and the biomonitoring reports from the Centers for Disease Control and Prevention have become key resources for research on human exposures to environmental chemicals, Europe has lagged behind. Following international policy decisions to decrease global mercury pollution, such human biomonitoring studies will be crucial to monitor the effects of the interventions.

## Conclusions

Annual benefits of removing Hg exposure can be estimated to be approximately €9 billion in Europe. While our results support enhanced public policies for the prevention of MeHg exposure, the economic estimates are highly influenced by uncertainties regarding the dose-response relationship. Thus, a logarithmic response curve results in 4-fold higher benefit estimates. In addition, benefits might be underestimated because costs linked to all aspects of neurotoxicity and long-term disease risks have not been considered. These European data and the calculated economic benefits support the need for interventions to minimize exposure to this hazardous pollutant.

## Abbreviations

DRF: Dose-response Function; EPA: Environmental Protection Agency; EU: European Union; GDP: Gross Domestic Product; hair-Hg: Mercury concentration in hair; MeHg: Methylmercury; IQ: Intelligence Quotient; perc: Percentile; PPP: Purchasing Power Parity; prob: Probability; RfD: Reference Dose; US: United States; WHO: World Health Organization.

## Competing interests

PG is an editor of this journal but did not participate in the editorial handling of this manuscript. The authors declare that they have no competing interests.

## Authors’ contributions

MB, CP, EBJ and PG planned the economic evaluation, carried out the calculations, and drafted the manuscript. AM reviewed published data on MeHg exposure. DA coordinated the contributions of the 17 DEMOCOPHES countries. AC and ME were responsible for the development and follow-up of the Standard Operating Procedures and Quality Assurance for hair sampling and mercury analyses in support to comparability of DEMOCOPHES measurements. DA, MB2, AC, MČ, PC, FD, MEF, AEG, KH, AK, LEK, MK-G, GK, DL, AM, MFR, PR, JST, and PW contributed unpublished exposure data from European countries and act as guarantors of the data applied. All authors commented on the draft manuscript, and all authors read and approved the final version.

## Authors’ information

National guarantors of the DEMOCOPHES data are listed as co-authors. The DEMO/COPHES Consortium that established and tested harmonised human biomonitoring on a European scale (http://www.eu-hbm.info) also included Jürgen Angerer, Pierre Biot, Louis Bloemen, Ludwine Casteleyn, Milena Horvat, Anke Joas, Reinhard Joas, Greet Schoeters, and Karen Exley.

## Supplementary Material

Additional file 1 Conversion rates, 2008. Click here for file

Additional file 2 Exposure distributions. Click here for file

Additional file 3 IQ calculation spreadsheet. Click here for file
