# Supplementary material for: Economic benefits of methylmercury exposure control in Europe: Monetary value of neurotoxicity prevention
Source: Environ Health. 2013 Jan 7;12:3. doi: 10.1186/1476-069X-12-3 (PMC3599906; doi:10.1186/1476-069X-12-3)
Supplement: Additional file 1 — Conversion rates, 2008. [file 1476-069X-12-3-S1.docx]

**Additional file 1. Conversion rates and adjustment factors used, 2008**

| Country | | Euro exchange rate | GDP $_US_ppp (1) | (Country GDPpp/capita)/ USGDPpp/capita (2) | IQ unit value (€)  ppp adjusted (3) | IQ unit value (€)  GDPppp/capita adjusted (4) |
| --- | --- | --- | --- | --- | --- | --- |
| Austria | | 1 | 0.852 | 0.842 | 16,045 | 15,849 |
| Belgium | | 1 | 0.874 | 0.781 | 16,459 | 14,713 |
| Bulgaria | | 1.96 | 0.782 | 0.275 | 7,513 | 5,185 |
| Croatia | | 7.22 | 4.34 | 0.360 | 11,320 | 6,782 |
| Cyprus | | 1 | 0.73 | 0.631 | 13,747 | 11,885 |
| Czech Republic | | 24.94 | 14.3 | 0.566 | 10,798 | 10,661 |
| Denmark | | 7.46 | 8.01 | 0.794 | 20,220 | 14,947 |
| Estonia | | 1 | 0.549 | 0.438 | 10,339 | 8,251 |
| Faroe Islands | | 7.46 | 8.01 | 0.794 | 20,220 | 14,947 |
| Finland | | 1 | 0.918 | 0.778 | 17,288 | 14,644 |
| France | | 1 | 0.922 | 0.706 | 17,363 | 13,305 |
| Germany | | 1 | 0.812 | 0.787 | 15,292 | 14,812 |
| Greece | | 1 | 0.701 | 0.610 | 13,201 | 11,484 |
| Hungary | | 251.51 | 129.429 | 0.416 | 9,691 | 7,838 |
| Ireland | | 1 | 0.952 | 0.919 | 17,928 | 17,306 |
| Italy | | 1 | 0.789 | 0.662 | 14,858 | 12,460 |
| Latvia | | 0.7 | 0.43 | 0.412 | 11,568 | 7,761 |
| Lithuania | | 3.45 | 1.77 | 0.390 | 9,662 | 7,346 |
| Luxembourg | | 1 | 0.906 | 1.676 | 17,062 | 31,570 |
| Malta | | 1 | 0.59 | 0.544 | 11,111 | 10,242 |
| Netherlands | | 1 | 0.842 | 0.886 | 15,857 | 16,692 |
| Norway | | 8.22 | 8.752 | 1.128 | 20,051 | 21,246 |
| Poland | | 3.51 | 1.86 | 0.383 | 9,979 | 7,207 |
| Portugal | | 1 | 0.649 | 0.512 | 12,222 | 9,650 |
| Romania | | 3.68 | 1.6 | 0.258 | 8,188 | 4,857 |
| Slovakia | | 1 | 0.533 | 0.474 | 10,037 | 8,936 |
| Slovenia | | 1 | 0.634 | 0.632 | 11,939 | 11,904 |
| Spain | | 1 | 0.72 | 0.659 | 13,559 | 12,406 |
| Sweden | | 9.62 | 8.77 | 0.798 | 17,168 | 15,019 |
| Switzerland | | 1.59 | 1.549 | 0.912 | 18,346 | 17,184 |
| United Kingdom | | 0.8 | 0.651 | 0.7915 | 15,325 | 14,906 |
| EU (27)^2^ | | 1 | 0.741 | 0.661 | 13,579 | 12,441 |
| US | | 1.47 | 1 |  |  |  |
|  |  | Source: Eurostats 2012, OECD 2012, Federal Reserve Bank of St Louis | | | | |

1. 2008 GDP $ Purchasing power parity (ppp) adjustment factor is used to eliminate price level differences between each country and the US (see details below).
2. $\frac{(GDPppp/capita)i}{GDPppp/capita)USA}$ with i for each country is the ratio of ppp-adjusted real GDP per capita in each country and the US one
3. $_2008_18,832 per IQ point was the US benefit value in 2008 (38). Using the adjustment factor (1), the value of each IQ point in €2008 is given per country after currency exchange for non-Euro member states (27)
4. Difference in each country and US incomes could increase or decrease the value of an IQ point. The estimate (4) adjusts the IQ value estimate by the ratio of ppp-adjusted real GDP/capita in each country and the US one. The estimated value of an IQ point is derived from the impact of labor costs & productivity.

Purchasing power parity or parities.

“Spatial deflators and currency converters, which eliminate the effects of the differences in price levels between countries, thus allowing volume comparisons of GDP components and comparisons of price levels.” PPPs are calculated in three stages: first for individual products, then for groups of products or basic headings and, finally, for groups of basic headings or aggregates. The PPPs for basic headings are unweighted averages of the PPPs for individual products. The PPPs for aggregates are weighted averages of the PPPs for basic headings. The weights used are the expenditures on the basic headings. PPPs at all stages are price relatives. They show how many units of currency A need to be spent in country A to obtain the same volume of a product or a basic heading or an aggregate that X units of currency B purchases in country B. In the case of a single product, the “same volume” means “identical volume”. But in the case of the complex assortment of goods and services that make up an aggregate such as GDP. The “same volume” does not mean an “identical basket of goods and services”. The composition of the basket will vary between countries according to their economic, social and cultural differences, but each basket will provide equivalent satisfaction or utility. Also referred to as “parity” or “parities” (Source, OECD Glossary).

EU Member states (27): Austria, Belgium, Bulgaria, Cyprus, Czech Republic, Denmark, Estonia, Finland, France, Germany, Greece, Ireland, Italy, Hungary, Latvia, Lithuania, Luxembourg, Malta, Netherlands, Poland, Portugal, Romania, Slovakia, Slovenia, Spain, Sweden, and United Kingdom.

Euro area member states (17) in 2008: Austria, Belgium, Cyprus, Estonia, Finland, France, Germany, Greece, Ireland, Italy, Luxembourg, Malta, Netherlands, Portugal, Slovakia, Slovenia, and Spain.
