# Supplementary material for: Economic benefits of methylmercury exposure control in Europe: Monetary value of neurotoxicity prevention
Source: Environ Health. 2013 Jan 7;12:3. doi: 10.1186/1476-069X-12-3 (PMC3599906; doi:10.1186/1476-069X-12-3)
Supplement: Additional file 2 — Exposure distributions. [file 1476-069X-12-3-S2.doc]

**Additional File 2. Distribution data on hair-mercury concentrations in European countries obtained from DEMOCOPHES and additional studies. Sources are as in Table 1.**

| Country | Median | 75^th^ percentile | 90^th^ percentile | 95^th^ percentile |
| --- | --- | --- | --- | --- |
|
| Austria | 0.118 | 0.215 | 0.417 | 0.636 |
| Belgium | 0.440 | 0.635 | 0.987 | 1.236 |
| Bulgaria* | 0.069 | 0.069 | 0.239 | 0.459 |
| Croatia | 0.604 | 0.954 | 1.810 | 2.480 |
| Cyprus | 0.485 | 0.730 | 1.515 | 2.065 |
| Czech Republic | 0.150 | 0.240 | 0.375 | 0.570 |
| Denmark | 0.435 | 0.680 | 1.115 | 1.508 |
| Estonia | 0.26 | 0.41 | 0.57 | 0.90 |
| Faroe Islands | 0.720 | 1.100 | 1.810 | 2.580 |
| Finland | 0.26 | 0.41 | 0.57 | 0.90 |
| France | 0.52 | 0.82 | 1.10 | 1.72 |
| Germany | 0.118 | 0.215 | 0.417 | 0.636 |
| Greece | 1.120 | 1.814 | 2.770 | 3.840 |
| Hungary | 0.045 | 0.113 | 0.194 | 0.279 |
| Ireland | 0.184 | 0.321 | 0.601 | 0.753 |
| Italy | 0.780 | 1.283 | 2.008 | 2.626 |
| Latvia | 0.26 | 0.41 | 0.57 | 0.90 |
| Lithuania | 0.26 | 0.41 | 0.57 | 0.90 |
| Luxembourg | 0.39 | 0.68 | 1.30 | 1.64 |
| Malta | 0.780 | 1.283 | 2.008 | 2.626 |
| Netherlands | 0.377 | 0.565 | 0.855 | 1.071 |
| Norway | 0.418 | 0.637 | (0.854) | 1.075 |
| Poland | 0.147 | 0.217 | 0.317 | 0.417 |
| Portugal | 1.237 | 1.712 | 2.459 | 2.981 |
| Romania* | 0.069 | 0.069 | 0.239 | 0.459 |
| Slovakia | 0.120 | 0.222 | 0.370 | 0.595 |
| Slovenia | 0.292 | 0.518 | 0.894 | 1.404 |
| Spain | 1.80 | 2.78 | 3.50 | 4.40 |
| Sweden | 0.26 | 0.41 | 0.57 | 0.90 |
| Switzerland | 0.190 | 0.333 | 0.515 | 0.692 |
| United Kingdom | 0.160  0.465 | 0.28  0.630 | 0.39  0.831 | 0.43  1.005 |

*78.3% of the observations (from Romania) were below the level of detection (0.137 µg/g) and were therefore assigned the same value of half of this level.
